# Genome Sequence of Fusarium graminearum Strain MDC_Fg1, Isolated from Bread Wheat Grown in France

**DOI:** 10.1128/MRA.01260-18

**Published:** 2018-11-15

**Authors:** Tarek Alouane, Hélène Rimbert, Francis Fabre, Florence Cambon, Thierry Langin, Ludovic Bonhomme

**Affiliations:** a INRA, UMR 1095, Genetics, Diversity and Ecophysiology of Cereals, Clermont-Ferrand, France; b Université Clermont Auvergne, INRA, Laboratoire de Génétique, Diversité et Ecophysiologie des Céréales, Clermont-Ferrand, France; Vanderbilt University

## Abstract

Fusarium graminearum is a major fungal pathogen that induces Fusarium head blight (FHB), a devastating disease of small-grain cereals worldwide. This announcement provides the whole-genome sequence of a highly virulent and toxin-producing French isolate, MDC_Fg1.

## ANNOUNCEMENT

Fusarium graminearum (phylum *Ascomycota*, class *Sordariomycetes*, order *Hypocreales*) is the main causal agent of the Fusarium head blight (FHB) disease that threatens small-grain cereals, including three of the world's four most widely produced cereals (i.e., maize, wheat, and barley). FHB induces severe losses in yield and grain quality and is of upmost concern for human and animal health because of the production and accumulation of mycotoxins such as deoxynivalenol (DON) (1). This makes F. graminearum among the 10 most-studied fungal pathogens in molecular plant pathology (2). Its whole-genome sequence was primarily made available and annotated from the North American strain PH-1 (3, 4) and further supplemented by other genomes obtained from Australian or European strains (5–7). Here, we present the whole-genome sequence of the MDC_Fg1 strain, also known as Fg1. MDC_Fg1 was isolated during field prospecting in the North of France and was selected from among a dozen isolates for its severe aggressiveness and its ability to produce DON (8, 9).

The fungus was grown on potato dextrose broth and the genomic DNA was prepared from the mycelium using a NucleoBond kit (Macherey-Nagel, Düren, Germany). Libraries were produced using a SMRTbell template prep kit 1.0 (Pacific Biosciences, Menlo Park, CA, USA). A size selection of 12 kb was realized by using the BluePippin system (Sage Scientific, Beverly, MA, USA) according to the protocol “20 kb Template Preparation Using BluePippin Size-Selection System” (Pacific Biosciences; see https://www.pacb.com/wp-content/uploads/Procedure-Checklist-20-kb-Template-Preparation-Using-BluePippin-Size-Selection-System-15-20-kb-Cutoff-Sequel-Systems.pdf). The genome of MDC_Fg1 was sequenced using the Sequel PacBio platform (Pacific Biosciences) and Sequel Sequencing kit 1.2. A total of 855,732 subreads were generated, with an *N*_50_ of 6,441 bp, which led to a total of 3,831,551,101 bases with a 74.37× mean coverage. The read sequences were filtered and assembled using the Hierarchical Genome Assembly Process (HGAP4) implemented in SMRT Link v5.0 (Pacific Biosciences) using default settings, which keeps only subreads with read quality (rq) of ≥0.7. The assembly resulted in 96 contigs, with a genome size of 36,807,931 bp, a G+C content of 47.97%, an *N*_50_ value of 1,646,471 bp, and a maximum contig size of 3,411,897 bp. The completeness of the assembly was assessed using Benchmarking Universal Single-Copy Orthologs (BUSCO) v3.0.2 (10), which estimated the genome assembly to be 98.2% complete, with 3,660 single-copy orthologs out of the 3,725 expected groups from the lineage *Sordariomycetes* data set.

### Data availability.

This whole-genome shotgun project has been deposited at DDBJ/ENA/GenBank under the accession number UIHA00000000 (BioSample accession number SAMEA4778736 and BioProject accession number PRJEB27611). The PacBio reads are available in SRA under accession number ERP109716. The version described in this paper is the first version, UIHA01000000.
